# Alien invasive *Leucaena leucocephala* successfully acquires nutrients by investing in below-ground biomass compared to native *Vachellia nilotica* in nutrient-amended soils in South Africa

**DOI:** 10.1093/aobpla/plac026

**Published:** 2022-05-26

**Authors:** Khululwa Ndabankulu, Zivanai Tsvuura, Anathi Magadlela

**Affiliations:** School of Life Sciences, University of KwaZulu-Natal, Westville Campus, Private Bag X54001, Durban 4000, South Africa; Centre for Functional Biodiversity, School of Life Sciences, University of KwaZulu-Natal, Pietermaritzburg Campus, Private Bag X01, Scottsville 3209, South Africa; School of Life Sciences, University of KwaZulu-Natal, Westville Campus, Private Bag X54001, Durban 4000, South Africa

**Keywords:** *Burkholderia*, emerging alien invasive, *Leucaena leucocephala*, P deficiency, soil acidity, *Vachellia nilotica*

## Abstract

Soils in grasslands and savannas of southern Africa are acidic and nutrient-poor. Legume plants, such as *Vachellia nilotica* and alien invasive *Leucaena leucocephala*, are a major component of the vegetation there. *Vachellia nilotica* can establish in drought-prone environments, and is invasive in high rainfall areas. *Leucaena leucocephala* is an emerging invasive in South Africa and is ranked among the world’s 100 most invasive alien species. Alien plants can invade native habitats through their adaptability to low-resource soils, and thus can out-compete and displace native vegetation. We investigated the effects of phosphorus (P) deficiency and soil acidity on legume–microbe symbiosis, nitrogen (N) nutrition and carbon (C) growth costs of these two legumes in grassland soils. We used as inoculum and growth substrate soils collected from a long-term (>65 years) nutrient and lime-addition trial, the Veld Fertilizer Trial (VFT), located at Ukulinga Research Farm near Pietermaritzburg in South Africa. We used soils from three VFT treatments: soils fertilized with superphosphate (336 kg ha^−1^) applied once per year (+P), soils fertilized with superphosphate (336 kg ha^−1^) applied once per year with dolomitic lime (2250 kg ha^−1^) applied once every 5 years (P+L) and soils with no superphosphate and no dolomitic lime applications (Control). Seeds of *V. nilotica* and *L. leucocephala* were germinated and grown independently in these soils in green house conditions and harvested after 125 days for measurement of growth, legume–microbe symbiosis, N nutrition and C growth costs. Results showed that the two legumes had different growth adaptations. *Vachellia nilotica* grown in control soils and +P soils nodulated with various *Burkholderia* spp., while *L. leucocephala* did not nodulate in all soil treatments. Both legumes utilized for growth both atmospheric- and soil-derived N across all treatments thereby decreasing C growth costs. *Vachellia nilotica* grown in +P soils accumulated the most biomass and N nutrition. *Leucaena leucocephala* maximized specific N assimilation rates by investing in below-ground biomass accumulation in control soils. This shows that *L. leucocephala* possesses traits that are successful in acquiring nutrients by investing in below-ground biomass and relying on utilization of N from both the soil and the atmosphere.

## Teaser

Alien plants such as *L. leucocephala*can invade native habitats through their adaptability to low-resource soils, and thus can out-compete and displace native vegetation. They do this by investing on their below-ground biomass, reliance in free-living N-fixing bacteria and altering N sources. Therefore, soil management practices that seek to address nutrient deficiency and soil acidity in nutrient-stressed ecosystems may affect the interaction between native and alien invasive woody plants in impacted ecosystems.

## Introduction

The characteristic acidity and limiting amounts of nutrients in soils from grassland ecosystems in South Africa (Craine *et al.* 2008) are a hindrance to plant growth and may affect plant species diversity (Sulieman and Tran 2015). Deficiency in amounts of phosphorous (P) and nitrogen (N) in soils constrain primary production in many terrestrial and aquatic ecosystems (Harpole *et al.* 2011). Mkhabela (2006) reported similar low soil nutrient conditions in crop production areas of the Midlands of KwaZulu-Natal province of South Africa, which were dominated by grasslands prior to large-scale disturbances of agriculture and human settlements. At molecular level, both nutrients are constituents of genetic material (i.e. DNA and RNA), protein (enzymes)/amino acids and also assist in the regulation of a multitude of bioenergetic processes (Duan *et al.* 2008). Phosphate is also found in the energy molecule adenosine triphosphate (ATP) that is involved in plant metabolic reactions, especially in legume plants since they require it for biological nitrogen fixation (BNF) (Rotaru and Sinclair 2009; Mitran *et al.* 2018). Some legumes and N-fixing bacteria may establish a symbiotic relationship that results in BNF. However, BNF requires high amounts of P as ATP (Rotaru and Sinclair 2009), which is utilized by microbes in nodules for metabolic pathways during dinitrogen (N_2_) reduction (Vance *et al.* 2003). Although N_2_ is abundant in the atmosphere (ca. 78 %), N deficiency in soils remains common (Zahran 1999; Ferguson *et al.* 2010). Plant root colonization by N-fixing microbes and nodule formation enables plants to tap into the N_2_ reservoir in the atmosphere and convert it into plant usable forms such as ammonia (NH_3_) and nitrate (NO_3_^−^) (Herridge *et al.* 2005; Sprent 2007). The BNF process is therefore important for modern day agriculture as it reduces the dependence on expensive and environmentally unfriendly chemical fertilizers (Zahran 1999; Herridge *et al.* 2005). For instance, the use of chemical fertilizers and their leaching contributes to algal blooms in aquatic ecosystems (Glibert *et al.* 2014, 2020). However, BNF is highly affected by a number of factors including P deficiency (Vance *et al.* 2003; Sulieman and Tran 2015).

Phosphorus is considered the second most limiting nutrient after N for plant growth (Olivera *et al.* 2004). Legume plants require up to 20 % of total plant P for nodule growth and function during atmospheric N fixation (Valverde *et al.* 2002; Olivera *et al.* 2004). However, P deficiency may affect the supply of carbon to the nodules so that bacteria will have higher respiratory demands during N fixation (Sar and Israel 1991; Valentine *et al.* 2011). Vardien *et al.* (2014) and Magadlela *et al.* (2014, 2015, 2016) showed that P deficiency increased nodule construction costs and growth respiration in *Virgilia divaricata*, which ultimately reduced nodulation and plant biomass. Low P availability is commonly reported in acidic soils (Balemi and Negisho 2012) because the P is fixed by cations such as aluminium (Al^3+^), iron (Fe^+3^) and manganese (Mn^2+^) thereby rendering it unavailable to plants (Chen and Liao 2016). Due to this, soil acidity is likely to affect nodulation and N fixation (Mohammadi *et al.* 2012; Bekere *et al.* 2013). However, the application of lime ameliorates soil acidity, thus increasing available soil P for plant uptake (Fageria and Baligar 2008; Holland *et al.* 2018). High P levels promote growth, productivity, nodulation and N fixation in legumes (Grant *et al.* 2001; Zaidi *et al.* 2009). Crawley *et al.* (2005) showed an interaction between lime and nutrients in determining productivity and species richness in grasslands, whereby the addition of lime in the soil resulted in increased plant growth. Goulding (2016) also provided a specific example of the benefits to productivity of liming upland grassland soils using research that began in the 1970s: livestock numbers doubled within 4 years of lime application because growth and productivity of clover pastures improved with quality produce for over 20 years. However, we know of no studies that have been conducted to determine the growth and nitrogen nutrition of invasive legumes grown in limed soils. A long-term field experiment on influences of fertilizer and lime application on grassland productivity at Ukulinga in South Africa presents an opportunity for testing relevant ecological questions on influences of addition of nutrients (N and P) and lime on grassland dynamics and functioning.

Although growth may be constrained, legume plants have metabolic and morphological adaptations that enable them to survive in acidic and P-deficient soils (Vance *et al.* 2003; Barea *et al.* 2005). In particular, Magadlela *et al.* (2014) report that the primary adaptation is the shift from N derived from the atmosphere to assimilation of soil N by roots. The shift of N sources is indicated by specific absorption and assimilation rates, as well as a reduction in BNF efficiency (Magadlela *et al.* 2016). Legume plants growing in P-limited soils have also been found to modify root morphology and increase root biomass, which increases the surface area of the roots for nutrient uptake (Li *et al.* 2016; Iqbal *et al.* 2020). In particular, native plants may allocate more biomass below ground (higher root mass ratio and root depth), which affords them greater access to water and nutrients compared to alien invasive species (Huang and Fry 1998; Franco *et al.* 2011). Associations of legumes with arbuscular mycorrhizal (AM) fungi, N-fixing bacteria and phospho-bacteria can increase plant nutrient assimilation rates and promote growth and productivity in nutrient-limited environments (Bilal *et al.* 2018; Qin *et al.* 2020). Even though legumes in symbioses with rhizobacteria may be involved in fixation of atmospheric N in P-deficient soils, the legumes can also assimilate available soil inorganic N to conserve energy (Mortimer *et al.* 2013; Magadlela *et al.* 2016).

Invasion by alien plants can occur in a range of native habitats including nutrient-poor environments where the invasive plants may effectively compete with native plants (Funk and Vitousek 2007; Sardans *et al.* 2017). Invasive legumes can more efficiently exploit scarce nutrients and yield greater amounts of above-ground biomass rich in N efficiently than their co-occurring native species. There is evidence that some invasive species may nodulate more readily and fix greater amounts of N than co-occurring N-fixing species (Rodriguez-Echeverria *et al.* 2009). Consequently, N increases beyond the levels in which indigenous species are adapted to thrive (Stock *et al.* 1995; Marchante *et al.* 2009) and may be displaced by invasive species due to their weedy growth (Mortimer *et al.* 2013). The amount of N contributed by BNF in native legumes is lower than that produced by invasive legumes, which can be attributed to slower growth rates and greater competition from natural enemies in the native plants (Blackburn *et al.* 2011). This may result in invasive species out-competing native species. Thus, invasive legumes may have adaptive traits or mechanisms that enhance their competitive ability for nutrient uptake compared to indigenous plants (Morris *et al.* 2011).

Hence, this study aimed to determine the effect of soil acidity on root microbial symbiosis, N nutrition, C construction costs and growth physiology of an indigenous and alien invasive legume collected from long-term Veld Fertilization Trial plots at Ukulinga Research Farm, near Pietermaritzburg in South Africa. It was hypothesized that soil acidity coupled with P stress would promote below-ground biomass of the alien invasive legume species (*Leucaena leucocephala*) resulting to increasing nutrient assimilation rates compared to *Vachellia nilotica*.

## Materials and Methods

### Study species and soil site

*Vachellia nilotica* (Fabaceae: Mimosoideae), formerly called *Acacia nilotica*, is a relatively common mimosoid tree species native to savannas and grasslands from southern Africa through East Africa to South East Asia (Beukes *et al.* 2019). The species is a small- to medium-sized tree that is semi-deciduous and invasive in high rainfall areas (Boon 2010). *Leucaena leucocephala* (Fabaceae: Mimosoideae) is a shrub or small tree that originates from Central America but has spread to many parts of the tropics, including southern Africa, where it is invasive and largely occurs in low altitude, disturbed areas (Boon 2010). The species is listed among 100 most invasive alien species in the world (Lowe *et al.* 2000).

Soil samples for this study were collected from the Veld (≈Field) Fertilizer Trial (VFT) located at the Ukulinga Research Farm (29°24ʹE, 30°24ʹS) near Pietermaritzburg in South Africa. The VFT site is located at an elevation of 838–847 m above sea level. The mean annual rainfall of approximately 838 mm largely falls in summer (October–April). The mean monthly minimum and maximum temperatures are 9 °C in July and 26 °C in February, respectively (Ward *et al.* 2020). The site has acidic, leached sandy soils derived from shales of the Karoo sedimentary sequence, and has significant amounts of clays derived from dolerite (Mucina and Rutherford 2006). The vegetation is described as KwaZulu-Natal Hinterland Thornveld of the sub-escarpment savanna (Mucina and Rutherford 2006), which at Ukulinga is a tall grassland consisting of the grasses *Hyparrhenia hirta*, *Panicum maximum*, *Themeda triandra* and *Tristachya leucothrix* with scattered *V. nilotica* and *V. sieberiana* trees.

### Experimental soil and soil nutrient analysis

Soils used as inoculum and growth substrate in this study were collected from the VFT, a long-term nutrient addition experiment that was initiated in 1951 through the manipulation of N, P and lime (L). The main objective of the VFT was to determine the effect of nutrient addition (N and P) and lime application on the production, crude protein content and composition of the grassland (Morris and Fynn 2001; Tsvuura and Kirkman 2013). The VFT initially (1951–2019) comprised of 96 plots each 9.0 m × 2.7 m in size with 1-m spacing between plots. The experiment was replicated in three blocks of 32 plots each. Treatments consisted of four independent variables consisting of two types of N fertilizer—limestone ammonium nitrate and ammonium sulphate each applied at four levels (control; 7.1; 14.1; 21.2 g m^−2^); P fertilizer applied at two levels (control; 33.6 g m^−2^); and lime applied at two levels (control; 225 g m^−2^) (see Tsvuura and Kirkman 2013). The design of the VFT was modified in 2019 so that each plot was subdivided into two subplots of 4 m × 2.7 m, with a 1-m wide buffer between them. In one subplot, the original treatment is applied, while the other subplot is not treated in order to examine the effects of reversal of the original treatment (K. Kirkman, pers. comm., School of Life Sciences, University of KwaZulu-Natal, Pietermaritzburg Campus, Private Bag X01, Scottsville 3209, South Africa). From the three blocks of the VFT, three treatments were used for this study: (i) subplots fertilized with superphosphate applied once per year (+P), (ii) subplots fertilized with superphosphate applied once per year coupled with application of dolomitic lime once every 5 years (P+L) and (iii) subplots not fertilized and not limed (i.e. no P+ no L; control). From each treatment subplot (hereafter referred to simply as plot), five soil samples (0–10 cm depth) were collected and pooled. Thereafter, three subsamples of 50 g of soil from each treatment were collected and sent for analysis of amounts of P, N, potassium (K), pH (KCl), exchangeable acidity and total cations at the Analytical Services Unit of the KwaZulu-Natal Department of Agriculture and Rural Development at Cedara, South Africa and microbial analysis. The analysis showed that P+L soils had a significantly higher P concentration, followed by +P soils compared to −P−L soils that had lowest P concentration **[see****Supporting Information—Table S1****]**. N concentration under +P soils was observed to be higher, while in −P−L soils the N concentration was lower. There was no significant difference in N and organic C concentrations among all treatments **[see****Supporting Information—Table S1****]**. P+L showed a significantly higher exchangeable acidity from the other soil treatments **[see****Supporting Information—Table S1****]**. pH level values showed that +P and −P−L soils were the most acidic and the least acidic soils were P+L **[see****Supporting Information—Table S1****]**. The most abundant bacterial genera identified in soils belonged to genus *Pseudomonas*. Different strains from the genus *Pseudomonas* were observed in all soil Veld Fertilization Trials at Ukulinga Farm. Following *Pseudomonas* spp., *Burkholderia contaminans* strain and *Sphingomonas* spp. N-9 were able to tolerate nutrient variability in these VFT soils. *Caulobacter rhizosphaerae* was only unique to low −P−L soils.

### Seed germination and growth conditions

Seeds of *V. nilotica* were collected from the Mposhini Nature Reserve near Pietermaritzburg and those of *L. leucocephala* were collected from the Roosenfontein Reserve near Durban, both in South Africa. The seeds were then scarified by soaking in 5 % (v/v) sodium hypochlorite for 20 min and then rinsed 10 times with distilled water. Thereafter, seeds were germinated in Petri dishes on filter paper moistened with distilled water. After seed germination, seedlings were planted 1.5 cm deep in greenhouse pots (15 cm diameter at top; 10 cm diameter at base; 12 cm height) with perforated bases, which were three-quarter filled with soil collected from the VFT. Each soil treatment had 20 replicate plants, which were grown in a greenhouse. Greenhouse conditions consisted of night-time and day-time temperatures of 12–14 °C and 30–35 °C, respectively. The humidity ranged from 70 to 80 % and the irradiance was ca. 35 % of full sunlight (415.6 μmol m^2^ s^−1^). Plants were watered every 2 days in the afternoon depending on the weather conditions.

### Plant harvest and nutrient analysis

Thirty days after seed germination, three plants per treatment were harvested for measurement of initial plant dry weight and N content. The plants were rinsed with tap water and oven-dried at 60 °C for 48 h. Plants were then separated into leaf, stem and root parts and the dry weights (DW) recorded.

After 125 days of growth, five plants per treatment were harvested and the same procedure of oven-drying, separation into parts and weighing carried out. The dry plant material was pulverized in a pestle and mortar and analysed for C, N, P and standard corrected δ^15^N concentrations. δ^15^N concentration analysis was conducted using a LECO nitrogen analyser at the Archaeometry Department, University of Cape Town, South Africa, and C and P concentrations were analysed using the inductively coupled mass spectrometry at the Central Analytical Facilities, Stellenbosch University, South Africa.

From the remaining plants, root nodules were harvested for bacterial extraction. Root nodules were rinsed with distilled water, then sterilized in 70 % (v/v) ethanol for 30 s and with 3.5 % (v/v) sodium hypochlorite solution for 3 min. Thereafter, nodules were rinsed 10 times with distilled water and put for storage in airtight vials containing silica gel and cotton wool. The vials were stored at 4 °C before bacterial extraction, culturing and sequencing.

### Bacterial extraction and identification

Prior to bacterial extraction, the nodules were transferred into 2-mL Eppendorf vials containing distilled water and left overnight to absorb water. The nodules were then sterilized in 70 % (v/v) ethanol for 30 s and with 3.5 % (v/v) sodium hypochlorite solution for 3 min and thereafter rinsed 10 times with distilled water. Nodules were then crushed in 15 % glycerol solution. The turbid nodule solution was then streaked in plates containing yeast mannitol agar (YMA) containing 0.5 g L^−1^ yeast extract (Biolab), 10 g L^−1^ mannitol (Saarchem), 0.5 g L^−1^ di-potassium hydrogen orthophosphate (K_2_HPO_4_, Biolab), 0.2 g L^−1^ magnesium sulphate heptahydrate (MgSO_4_·7H_2_O, Biolab), 0.1 g L^−1^ sodium chloride (NaCl, Biolab), 15 g L^−1^ bacteriological agar (Biolab) and incubated at 28 °C for 4 days. The bacteria were re-streaked into fresh plates until pure colonies were obtained.

The pure bacterial colonies were amplified using a portion of 16S rRNA gene, 27F (5ʹ-AGAGTTTGATCCTGGCTCAG-3ʹ) and 1492R (5ʹ-GGTTACCTTGTTACGACTT-3ʹ). Bacterial DNA amplification was conducted on a BioRad Mini Opticon Thermal cycler (BioRad, South Africa) using the following protocol: initial denaturation for 5 min at 94 °C, 30 cycles of denaturation for 30 s at 94 °C, annealing at 55 °C and elongation for 2 min at 72 °C, followed by a final elongation step of 10 min at 72 °C. Each 25 µL PCR reaction volume contained 11 µL sterile distilled water, 12.5 μL TAKARA-EmeraldAmpGT PCR Master Mix (Separations, South Africa), 0.25 µL 27F primer, 0.25 µL 1492R primer and 1 µL of DNA colony. The results were viewed with gel electrophoresis (1 % agarose gel using TAE buffer). The PCR products were sent for sequencing at the Central Analytical Facility. The resulting sequences were edited and subjected to BLASTN searches (National Center for Biotechnology Information) NCBI (https://www.ncbi.nlm.nih.gov).

### Calculations of growth, carbon construction costs and N assimilation and utilization rates

Values of root-to-shoot ratio (R:S) were obtained from calculating the root dry weight per shoot dry weight of the plant. The relative growth rate (RGR) was calculated according to Ågren and Franklin (2003) as RGR = [(ln *W*2 − ln *W*1)/(*t*2 − *t*1)], where *W*2 − *W*1 represent plant dry weight accumulated from the initial harvest to the final harvest and *t*2 − *t*1 is the time (in days) between harvests.

Carbon construction costs (Cw) were calculated according to Mortimer *et al.* (2005), modified by Peng *et al.* (1993) as: Cw = (*C* + *kN*/14 × 180/24) (1/0.89) (6000/180), where Cw is the total carbon construction cost of the tissues [mmol C g^−1^ dry weight (DW)], *C* is the total concentration of C (mmol C g^−1^), *k* is the reduction state of the N substrate (for NH_3_ = −3) and *N* is the total organic N content of the tissue (g DW^−1^) as described by Williams (1987). The numerical value 14 is the atomic mass of N, 180 is a conversion factor from moles to grams of glucose and 24 is the number of electrons in a glucose molecule, while 0.89 is an estimate of growth efficiency (Williams *et al.* 1987). The fraction 6000/180 is a constant conversion factor from g^−1^ dry weight to mmol C g^−1^ DW for glucose.

Initial and final amounts of total plant N were used to calculate the specific N absorption rate (SNAR), which is the net N absorption rate per unit root DW (mg N g^−1^ root DW N day^−1^) as: SNAR = (*N*2 − *N*1)/(*t*2 − *t*1) * [(loge *R*2 − loge *R*1)/(*R*2 − *R*1)], where *N* represents the total N content in the plant. The duration of plant growth is represented by *t*2 − *t*1 and *R*2 − *R*1 is the accumulation of root dry weight (Nielsen *et al.* 2001).

The specific N utilization rate (SNUR) indicates the DW gained for the N taken up by the plant (g DW mg^−1^ N day^−1^) as: SNUR = (*W*2 − *W*1)/(*t*2 − *t*1) * [(loge *N*2 − loge *N*1)/(*N*2 − *N*1)], where *W*, *t* and *N* are as defined above.

### Calculation of percentage N derived from the atmosphere

Nitrogen isotope analysis was carried out at the Archeometry Department, University of Cape Town, South Africa. The isotopic ratio of N was calculated as δ = 1000(*R*_sample_/*R*_standard_), where *R* is the molar ratio of the heavier to the lighter isotope of the samples and standards. Each milled sample of 2.1–2.2 mg was weighed into 8 mm × 5 mm tin capsules (Elemental Microanalysis, Devon, UK) on a Sartorius microbalance (Göttingen, Germany). The samples were then combusted in a Fisons NA 1500 (Series 2) CHN analyser (Fisons Instruments SpA, Milan, Italy). The N isotope values for the N gas released were determined on a Finnigan Matt 252 mass spectrometer (Finnigan MAT GmbH, Bremen, Germany) which was connected to a CHN analyser by a Finnigan MAT Conflo control unit. Three standards were used to correct the samples for machine drift, namely, two in-house standards (Merck Gel and Nasturtium) and the IAEA (International Atomic Energy Agency) standard (NH_4_)_2_SO_4_. The percentage N derived from the atmosphere was calculated as: %NDFA = 100((δ^15^N reference plant − δ^15^N legume)/(δ^15^N reference plant − β)).

The β-value was the δ^15^N natural abundance of the N derived from biological N_2_ fixation.

### Data analysis

Dry weights, growth parameters, specific N absorption rates, specific N utilization rates, %NDFA, atmospheric-derived N, soil-derived N and were compared among the fertilizer and lime treatments using analysis of variance (ANOVA) in IBM SPSS Statistics for Windows v. 27 (IBM Corp. 2017). Where the ANOVA results showed significant differences (*P* < 0.05) among treatments, a Bonferroni *post hoc* test was used to separate the means.

## Results

### Identification of endophytic bacterial isolates

The 16S ribosomal RNA gene partial sequences of bacterial colonies obtained from root nodules of *V. nilotica* plants grown in +P and −P−L soils revealed that these plants established symbiosis with *Burkholderia*. Specifically, there was a 99.01 % sequence similarity between the bacteria from root nodules and *B. contaminans* strains J8A6SARS (MT 409575).

### Biomass and growth kinetics

Whole-plant biomass of *V. nilotica* and *L. leucocephala* saplings was significantly higher in +P soils than in P+L and control soils (Table 1). Similarly, the shoots and roots of *V. nilotica* attained significantly greater biomass in +P soils than in P+L and control soils. Shoot biomass of *L. leucocephala* was significantly higher in P+L than in control soils. Inversely, the root biomass of *L. leucocephala* saplings was significantly higher in control than in P+L soils (Table 1).

**Table 1.** Mean (±1 SE) dry weight and growth kinetics of 125-day-old *V. nilotica* (V.n.) and *L. leucocephala* (L.l.) plants grown in soils supplemented with phosphorus and lime, soils rich in phosphorus and acidic soils collected from Ukulinga farm, KwaZulu-Natal. Values are based on *n* = 5 samples. In each row, different superscript letters indicate significant differences (*P* < 0.05) among treatments based on the Bonferroni *post hoc* test.

Parameter	P+L		+P		−P−L (Control)	
	L.l.	V.n.	L.l.	V.n.	L.l.	V.n.
Dry weights (g)						
Whole plant	1.41 ± 0.99^a^	2.04 ± 0.24^b^	1.64 ± 0.12^b^	4.42 ± 0.34^c^	1.45 ± 0.21^a^	1.55 ± 0.09^ab^
Shoot	1.16 ± 0.94^a^	1.88 ± 0.22^b^	0.95 ± 0.06^ad^	2.41 ± 0.21^c^	0.46 ± 0.04^d^	1.43 ± 0.06^b^
Nodules	—	—	—	0.01 ± 0.01^a^	—	0.01 ± 0.01^a^
Root	0.26 ± 0.05^a^	0.17 ± 0.02^a^	0.69 ± 0.06^ac^	1.91 ± 0.11^b^	0.99 ± 0.17^c^	0.11 ± 0.02^a^
Growth kinetics						
Root:shoot ratio	1.41 ± 0.11^a^	2.04 ± 0.24^b^	1.64 ± 0.12^a^	4.40 ± 0.32^c^	1.96 ± 0.21^ab^	1.54 ± 0.08^a^
Relative growth rate (g day^−1^)	0.02 ± 0.001^a^	0.02 ± 0.002^a^	0.02 ± 0.001^a^	0.02 ± 0.001^a^	0.02 ± 0.001^a^	0.02 ± 0.002^a^
C costs (mmol C g^−1^ DW)	0.006 ± 0.0002^a^	0.007 ± 0.0005^a^	0.004 ± 0.0.0003^ab^	0.002 ± 0.0004^b^	0.005 ± 0.0002^a^	0.003 ± 0.0002^b^

Root:shoot ratio of *V. nilotica* saplings was highest in +P soils, and was significantly higher in P+L than in control soils. For *L. leucocephala* samplings, the root:shoot ratio was similar among the soils. Relative growth rate was similar between the tree species and among treatments. The C costs were significantly higher in P+L than in other soils for *V. nilotica* but was similar among soil treatments for *L. leucocephala* (Table 1).

### N-source preferences

Saplings of *V. nilotica* and *L. leucocephala* in all soil treatments showed reliance on N derived from both the atmosphere and the soil. However, all saplings had greater reliance on soil N than atmospheric N (Table 2). Both *V. nilotica* and *L. leucocephala* grown in P+L soils showed significantly higher amounts of N derived from the soil (NDFS) than plants grown in other soils. Plants of both species grown in +P soils had the lowest amount of N derived from the soil. Additionally, *V. nilotica* and *L. leucocephala* showed significantly higher amounts of N derived from the atmosphere (NDFA) in P+L than in control soils. Plants of both species grown in control soils had significantly low rates of N derived from the atmosphere (%NDFA) and showed about four times less assimilation than in +P soils (Table 2).

**Table 2.** Mean (±1 SE) values of N fixation parameters determined in *V. nilotica* (V.n.) and *L. leucocephala* (L.l.) plants grown for 120 days in soils supplemented with phosphorus and lime, soils rich in phosphorus and acidic soils. Values are expressed as means ± SE, based on *n* = 5 samples. In each row, different letters represent significant differences among treatments (*P* < 0.05) based on the Bonferroni *post hoc* test.

Parameter	P+L		+P		−P−L (Control)	
	L.l.	V.n.	L.l.	V.n.	L.l.	V.n.
Standard corrected N (^14^N/^15^N)	2.64 ± 0.26^a^	2.26 ± 0.13^a^	1.63 ± 0.29^b^	1.76 ± 0.31^b^	3.73 ± 0.06^c^	3.756 ± 0.23^c^
N derived from atmosphere (%)	24.18 ± 3.82^a^	28.81 ± 4.60^a^	38.81 ± 4.26^b^	37.01 ± 4.60^b^	8.47 ± 0.93^c^	8.040 ± 3.35^c^
Atmospheric N derived (mmol g^−1^)	1.12 ± 0.11^a^	1.46 ± 0.15^a^	1.03 ± 0.10^a^	0.91 ± 0.06^a^	0.22 ± 0.01^b^	0.208 ± 0.01^b^
Soil-derived N (mmol g^−1^)	3.52 ± 0.37^a^	3.43 ± 0.35^a^	1.62 ± 0.16^b^	1.57 ± 0.11^b^	2.41 ± 0.12^c^	2.384 ± 0.21^c^

### Mineral nutrition

**Figure 1.**
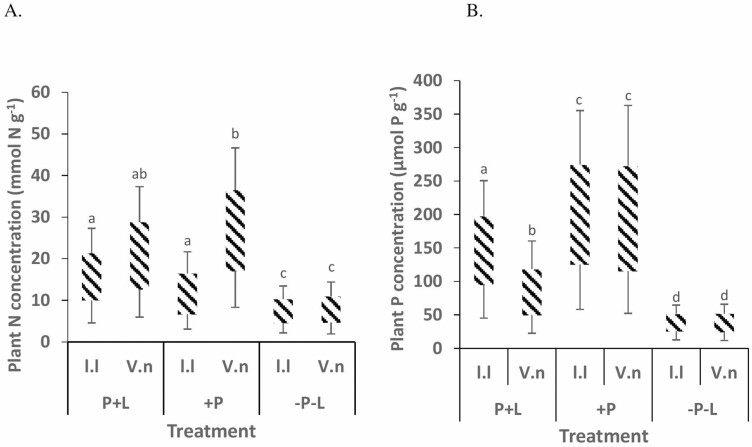
Mean (±1 SE) concentration (mmol P g^−1^) of plant (A) nitrogen and (B) phosphorus in *V. nilotica* and *L. leucocephala* grown for 125 days in soils supplemented with phosphorus and lime, soils rich in phosphorus and acidic soils collected at Ukulinga farm, South Africa. Different superscript letters indicate significant differences (*P* < 0.05) among treatments based on the Bonferroni *post hoc* test.

The N concentration of *V. nilotica* saplings was significantly higher in +P soils than in control soils (Fig. 1A), while the N concentration of *L. leucocephala* grown saplings was significantly higher in P+L soils than in control soils. The P concentration of both species was significantly higher in +P soils than in control soils (Fig. 1B). For both N and P, concentrations were also significantly lower in control soils than in P+L soils.

### Specific N assimilation and utilization rates

**Figure 2.**
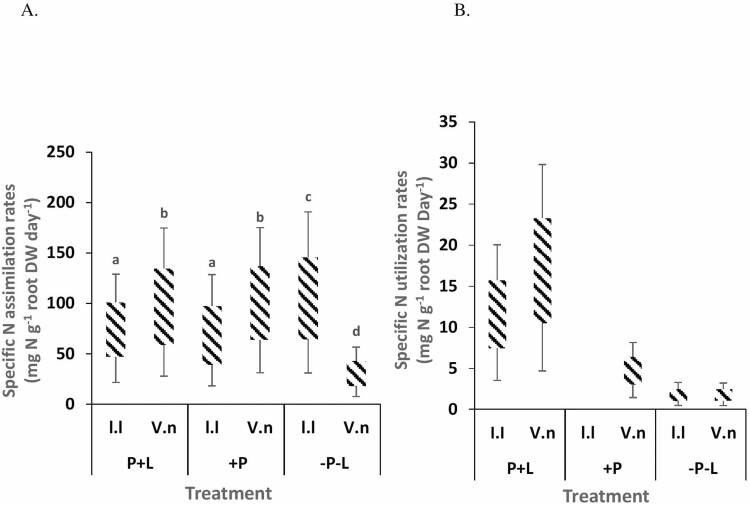
Specific N (A) assimilation and (B) utilization rates of *V. nilotica* and *L. leucocephala* saplings grown for 125 days in soils supplemented with phosphorus and lime, soils rich in phosphorus and acidic soils collected at Ukulinga farm, KwaZulu-Natal. Different superscript letters indicate significant differences (*P* < 0.05) among treatments based on the Bonferroni *post hoc* test.

Specific N assimilation rates (SNAR) of *V. nilotica* plants were significantly greater in P+L soil than in control soils. The SNAR of *L. leucocephala* plants was significantly higher in control soil than in soils of both P+L and +P (Fig. 2A). Specific N utilization rate of *V. nilotica* was different among all three soils, and was highest in P+L soils and lowest in control soils. For *L. leucocephala* saplings, SNUR was also significantly higher in P+L soils than in the other soils (Fig. 2B).

## Discussion

*Leucaena leucocephala* invested in below-ground biomass in control soils and the altered root architecture may have contributed to increased specific N assimilation rates. In acidic soils *Burkholderia* spp. were effective nodule inducers and contributed to the atmospheric N assimilation in *V. nilotica* saplings. Members of the β-rhizobium genus *Burkholderia* have been reported to form functional nodules with native *V. divaricata* growing in nutrient-deficient and acidic Cape Fynbos Mediterranean ecosystem soils (Magadlela *et al.* 2017). Also, other native Fynbos tree legumes such as *Psoralea*, *Hypocalyptus*, *Podalyria*, *Cyclopia* and *Virgilia oroboides* are reportedly nodulated by a variety of bacteria including *Burkholderia* spp. (Elliot *et al.* 2007; Kanu and Dakora 2012; Beukes *et al.* 2013). Not limited to the Cape Fynbos, Sanginga *et al.* (1996) reported similar findings regarding N_2_ fixation capabilities of *Burkholderia* spp. in symbiosis with *Mucuna pruriens* in savanna-type soils. Indigenous *M. pruriens* assimilated close to 40 % and more of N from the atmosphere in West African savanna-type soils (Sanginga *et al.* 1996). Additionally, *Burkholderia* spp., which can nodulate herbaceous legumes, have previously been reported by Garau *et al.* (2009) on a native legume herb *Rhynchosia ferulifolia*. The ability of the native legume, *V. nilotica*, to form a symbiotic relationship with *Burkholderia* spp. suggests that *Burkholderia* can withstand and function in low-nutrient and acidic soils as documented in previous studies (Kunito *et al.* 2012; Stopnisek *et al.* 2014; Lian *et al.* 2019).

Plants modify their root systems in stressful conditions to prevent senescence and mitigate the effects of stress (Franco *et al.* 2011). Enhanced root acquisition efficiency for nutrients has been identified as a critical coping strategy during P deficiency (Mortimer *et al.* 2008, 2009; Le Roux *et al.* 2009). This is consistent with our observations as *L. leucocephala* plants increase root dry weight and increased root surface area increases the probability that plant roots will contact more plant growth-promoting microorganisms and soil nutrients. Also, it has been reported that P deficiency reduces above-ground biomass and the inverse in below-ground biomass (Chaudhary *et al.* 2008). Our results concur with this as we observed a significant reduction in shoot biomass in *L. leucocephala* plants grown in acidic soils with high P stress, while their root biomass significantly increased, resulting in a higher specific N assimilation rate. The investment of alien invasive legume species (*L. leucocephala*) on below-ground biomass compared to the native legume species (*V. nilotica*) may be a growth strategy by alien invasive legume plants to increase nutrient assimilation in low-resource soils, and thus can out-compete and displace native vegetation. Additionally, *L. leucocephala* may have established symbiosis with AM fungi in acidic soils; AM fungi can enhance nutrient uptake under nutrient-deficient conditions (De Jaeger *et al.* 2011). However, this was not analysed in the current study. It is also important to note that the low N concentration levels in the acidic soils could have facilitated the survival of AM fungi as fungal diversity and abundance tend to decline in high N soil environments (Patra *et al.* 2005). In contrast, Tsvuura *et al.* (2017) reported greater biomass of extraradical fungal hypha in the N-fertilized plots of the VFT, but acknowledged that this could have been driven by nitrophilous mycorrhizal species.

Despite the lack of nodulation in invasive *L. leucocephala* in all the soil treatments, it was still able to fix atmospheric N. The NDFA of *L. leucocephala* grown in acidic and P-rich soils was the highest compared to *V. nilotica*. This suggests that the free-living N-fixing bacteria identified in the VFT soils such as *Pseudomonas* spp., *Sphingomonas* spp. and *C. rhizosphaerae* may have been functional in fixing atmospheric N for plant assimilation without inducing nodulation. The non- nodulation reduces the costs required by symbionts from plant hosts (Lu *et al.* 2017; Martínez-Hidalgo and Hirsch 2017). P deficiency has been reported to affect the supply of carbon to the nodules as bacteria have higher respiratory demands during N fixation (Sar and Israel 1991; Valentine *et al.* 2011). Nodule construction cost and growth respiration in *V. divaricata* increased with P deficiency, reducing nodulation and plant biomass (Vardien *et al.* 2014; Magadlela *et al.* 2016). Legumes can rely on actinomycetes and gram-positive bacteria for N fixation without nodulation (Bhatti *et al.* 2017). Similarly, Matiwane *et al.* (2019) observed non-nodulation in *Vigna radiata* plants grown in grassland soils in KwaZulu-Natal region of South Africa. Further explanations for non-nodulation could be the soil’s high exchangeable acidity and low pH (Lin *et al.* 2012). Cation sequestered P in acidic soils is likely to affect nodulation and N fixation (Mohammadi *et al.* 2012; Bekere *et al.* 2013). The reliance of alien invasive legume such as *L. leucocephala* to free-living N-fixing bacteria may promote their adaptability to invade native nutrient-limited ecosystems.

*Vachellia nilotica* and *L. leucocephala* were able to utilize N from both atmospheric and soil sources. Even though assimilation rates from different sources were not calculated, it has been highlighted that it is less costly for plants to assimilate soil N than atmospheric N fixation during growth in nutrient-deficient soil environments (Hellsten and Huss-Danell 2000; Høgh-Jensen *et al.* 2002; Vance *et al.* 2003). This is supported by Pate *et al.* (1979) who showed that the legume lupin consumed 10.2 mg C/mg N fixed during BNF and 8.1 mg C/mg N for NO_3_^−^. A similar trend (8.28 mg C/mg N and 4.99 mg C/mg N, respectively) was observed by Finke *et al.* (1982) in soybean. Minchin and Witty (2005) explained that, although C costs imposed may be statistically insignificant or show minimal differences as observed in this study, the overall plant development may be significant.

## Conclusion

The overall growth of the alien invasive legume *L. leucocephala* was negatively affected by nutrient deficiency and soil pH status compared to native *V. nilotica*. However, *L. leucocephala* invested in below-ground biomass during acidic soils in order to maximize the surface area for nutrient acquisition through altered root architecture. This legume plant was able to maintain their growth by relying on both atmospheric- and soil-derived N across all treatments. The acquiring of N derived from atmospheric by unnodulated *L. leucocephala* highlights the significance of free-living N_2_-fixing bacteria in acidic and P-deficient soil ecosystems. The investment of alien invasive legume species (*L. leucocephala*) on below-ground biomass, reliance in free-living N-fixing bacteria and altering N sources to maintain its growth show that *L. leucocephala* possess traits that are successful in acquiring nutrient especially in nutrient-limited soil environments. These collective adaptations by this alien invasive legume species (*L. leucocephala*) and other alien invasives in low-resource ecosystem soils can promote their persistence, displacing native vegetation. Therefore, soil management practices that seek to address nutrient deficiency and soil acidity in nutrient-stressed ecosystems may affect the interaction between native and alien invasive woody plants in impacted ecosystems.

## Supporting Information

The following additional information is available in the online version of this article—

**Table S1.** Mean (± 1SE) concentration of soil nutrients, total cations, exchangeable acidity and pH in soils supplemented with phosphorus and lime, soils rich in phosphorus and acidic soils collected at Ukulinga farm, KwaZulu-Natal. Values are based on *n* = 5 replicates. In each row, different superscript letters represent significant differences among treatments (*P* < 0.05) based on Bonferroni post hoc test.

plac026_suppl_Supplementary_Tables

## Data Availability

The data and material can be made available upon request from the corresponding author Dr A.M. at anathimagadlela@icloud.com.

## Notes

**Plants, Ecosystems & Climate. Chief Editor**: Mary Heskel
