# Supplementary material for: Alien invasive Leucaena leucocephala successfully acquires nutrients by investing in below-ground biomass compared to native Vachellia nilotica in nutrient-amended soils in South Africa
Source: AoB Plants. 2022 May 26;14(3):plac026. doi: 10.1093/aobpla/plac026 (PMC9211186; doi:10.1093/aobpla/plac026)
Supplement: plac026_suppl_Supplementary_Tables [file plac026_suppl_supplementary_tables.pdf]

Supplementary table S1: Mean (± 1SE) concentration of soil nutrients, total cations, exchangeable acidity and pH in soils supplemented with phosphorus and lime, soils rich in phosphorus and acidic soils collected at Ukulinga farm, KwaZulu-Natal. Values are based on n = 5 replicates. In each row, different superscript letters represent significant differences among treatments (*P* < 0.05) based on Bonferroni post hoc test.

**Parameter**	P+L	+P	-P-L (Control)
**Soil nutrients**			
P (mmol/g)	0.29 ± 0.02^{a}	0.26 ± 0.01^{a}	0.08 ± 0.00^{b}
N (mmol/g)	0.20 ± 0.00^{a}	0.20 ± 0.00^{a}	0.19 ± 0.00^{a}
K (mmol/g)	4.22 ± 0.67^{a}	4.23 ± 0.68^{a}	2.53 ± 0.34^{b}
Organic C (mmol/g)	3.56 ± 0.05^{a}	3.71 ± 0.02^{a}	3.69 ± 0.17^{a}
Ca (mmol/g)	63.25 ± 1.77^{a}	35.16 ± 0.68^{b}	30.79 ± 1.54^{c}
Mg (mmol/g)	19.39 ± 1.88^{a}	15.19 ± 0.59^{b}	19.69 ± 1.19^{a}
**Relative acidity**			
Exchange acidity (cmol L^{-1})	0.42 ± 0.25^{a}	0.24 ± 0.05^{b}	0.13 ± 0.02^{c}
Total cations (cmol L^{-1})	25.91 ± 1.69^{a}	27.14 ± 1.30^{b}	27.08 ± 2.60^{c}
pH (KCl)	6.33 ± 0.06^{a}	4.65 ± 0.01^{b}	4.63 ± 0.03^{b}
